# Upcycling of Defatted Sesame Seed Meal via Protein Amyloid-Based Nanostructures: Preparation, Characterization, and Functional and Antioxidant Attributes

**DOI:** 10.3390/foods13142281

**Published:** 2024-07-20

**Authors:** Fredrick Nwude Eze, Rattana Muangrat, Sudarshan Singh, Wachira Jirarattanarangsri, Thanyaporn Siriwoharn, Yongyut Chalermchat

**Affiliations:** 1 Office of Research Administration, Chiang Mai University, Chiang Mai 50200, Thailand; fredrickeze10@gmail.com; 2 Faculty of Agro-Industry, Chiang Mai University, Chiang Mai 50100, Thailand; wachira.j@cmu.ac.th (W.J.); thanyaporn.s@cmu.ac.th (T.S.); yongyut.c@cmu.ac.th (Y.C.); 3 Department of Food Process Engineering, Faculty of Agro-Industry, Chiang Mai University, Chiang Mai 50100, Thailand; 4 School of Medical & Allied Sciences, K.R. Mangalam University, Gurugram 122103, India; sudarshansingh83@hotmail.com

**Keywords:** sesame seed protein isolate, sesame seed globulin, plant-based proteins, food protein amyloids, antioxidant activity, agri-food residue valorization

## Abstract

Herein, the possibility of valorizing defatted sesame seed meal (DSSM) as a viable source for valuable plant proteins and amyloid-based nanostructure was investigated. Sesame seed protein isolate (SSPI) and the major storage protein globulin (SSG) were prepared by alkaline extraction–isoelectric point precipitation as well as fractionation in the case of SSG. The protein samples were characterized for their physicochemical attributes. SSPI and SSG were also evaluated for their ability to form amyloid structures under heating (90 °C) at low pH (2.0). Additionally, the functional attributes, antioxidant activity, and biocompatibility of the proteins and amyloid nanostructures were also examined. SSPI and SSG were both successfully prepared from DSSM. The data showed that the physicochemical attributes of both protein samples were quite similar, except for the fact that SSG was mostly composed of 11S globulin, as evinced by Tricine-SDS-PAGE analysis. TEM micrographs revealed that SSG was able to form curly-shaped fibrillar amyloid structures, whereas those derived from SSPI were mostly amorphous. Thioflavin-T assay and Tricine-SDS-PAGE analysis indicated that acidic heating promoted protein hydrolysis and self-aggregation of the hydrolyzed peptides into a β-sheet rich amyloid structure. Importantly, the amyloid preparations displayed commendable solubility, superior water and oil holding capacities, and antioxidant activity against DPPH and ABTS. The protein amyloid nanostructures were found to be non-toxic against RAW264.7 cells, HaCaT cells, and red blood cells. These findings indicate that DSSM could be upcycled into valuable protein amyloid structures with good potentialities as novel food ingredients.

## 1. Introduction

Amyloid-based nanostructures have continued to command intense research interest due to their intriguing structure and versatile roles in areas as diverse as materials, biomedicine, and food science. Evidence emerging from the previous decade does not only demonstrate that food protein amyloid nanostructures are safe for human nutrition [1] but also indicates their potential multifarious applications as transporters for the delivery of valuable bioactive compounds and nutrients [2] and sustainable food coating materials [3,4], as well as in the development of various novel food products such as cultivated meat [5]. Food protein amyloid nanostructures, by virtue of their unique physicochemical attributes, have been found to enhance the solubility, thermal and photostability, and biological properties of various functional ingredients and food products [6,7]. Interestingly, it has also been noticed that protein amyloid nanostructures exhibit superior techno-functional characteristics and antioxidative attributes when compared to their precursor proteins [8,9], underscoring their potential importance in the modulation of food quality and design of food with desirable attributes. Nonetheless, widespread application remains elusive because animal proteins used as source materials are expensive. Thus, there is a clear need for cheaper, more abundant, and more sustainable sources of food proteins capable of producing amyloid nanostructures. In this regard, plant-derived protein appears to be very promising.

Recent investigations have largely been focused on the preparation and characterization of amyloid nanostructure from more conventional food protein sources such as whey [9], soy [5], rice [8], and oats [10]. The successful formation of protein amyloid-based nanostructures was accomplished through the process of aggregation facilitated by thermal-induced denaturation and polypeptide hydrolysis, often at temperatures around 80–95 °C and low pH (2.0). Less-conventional plant protein sources, such as amaranth protein-rich waste [11] and defatted hempseed [12], have also been explored. The challenge often encountered in using plant-derived proteins for amyloid formation is that they are complex and of low purity. In addition, the presence of small bioactive molecules in the protein concentrate or isolate may hamper amyloid fibril formation. The source of the plant protein and the preparation strategy may improve the outcome.

Defatted sesame seed meal (DSSM) is an interesting but unexplored resource that could be potentially utilized as a sustainable, cheap, and abundant plant protein source for amyloid-based nanostructures. DSSM is an agri-food industry residue obtained from the extraction of sesame seed oil. DSSM could also be used as organic nitrogenous fertilizer [13]. Remarkably, DSSF is exceedingly rich in proteins (33–40%, depending on the source) [14], and as a result, it is often used in feed formulation for cattle, poultry, and fish [15]. Sesame seed protein is mainly composed of four storage proteins, namely prolamin (1.3%), glutelin (6.9%), albumin (8.6%), and globulin (67.3%). In other words, globulin (conventionally called α-globulin) and, to a lesser extent, albumin (conventionally called β-globulin) make up the majority of the proteins in sesame seed, together accounting for more than 90% of sesame seed storage proteins [16,17]. This implies that a large amount of sesame seed globulin (SSG) could be obtained from DSSM without much difficulty. Additionally, sesame seed protein isolate (SSPI) obtained from DSSM is regarded as high quality and capable of meeting the essential amino acid requirement for adults, according to FAO/WHO (2007) [18]. SSPI is unique in its rich content of sulfur-containing amino acids (3.8–5.5%), especially methionine (2.5–4%) [17], making it a reliable complementary source for balancing most plant proteins, which are limited in these amino acids, such as soy and ground rice. Sesame protein and peptides have also exhibited commendable biological properties (antioxidative, antihyperlipidemic, antihypertensive) [19,20,21] and functionality (surface properties and hydration) [16], underscoring their potential in the development of food products for health improvement. DSSM is very cheap (0.27–0.41 USD/kg) and abundant [22]; thus, it can be an inexpensive, readily available, and sustainable source for not only valuable plant proteins but also plant protein amyloid nanostructures. At the moment, there is a paucity of reports on the preparation of food amyloid nanostructures from plant proteins. Particularly, there is no report on the preparation and characterization of protein amyloid nanostructure from proteins derived from DSSM.

Thus, this work is focused on investigating the viability of proteins derived from defatted black sesame seed meal (DSSM) as a source material for the production of plant protein amyloid-based nanostructures. To this end, sesame seed protein isolate (SSPI) was prepared from DSSM using the alkaline extraction–isoelectric point precipitation (AE-IP) method. Being the most abundant protein group, sesame seed globulin fraction (SSG) was also prepared from DSSM via alkaline extraction, fractionation, and the isoelectric point precipitation (AE-F-IP) method. The ability of both SSPI and SSG to form amyloid nanostructures under thermal-induced aggregation in acidic conditions (90 °C, pH 2.0) was investigated. Furthermore, the amyloid nanostructures from both protein samples were characterized, and their functional, antioxidant properties and biocompatibility were examined. The findings from this work are expected to open an additional frontier for the valorization of defatted black sesame seed meal in the food industry.

## 2. Materials and Methods

### 2.1. Materials

Black sesame seeds sourced from the Huai Siao Royal Project Development Center in Ban Pong Subdistrict, Hang Dong District, Chiang Mai Province, Thailand, were subjected to roasting at 80 °C prior to extraction using supercritical CO_2_ (at 45 °C and 225 bars for an extraction duration of 3 h). The resultant defatted black sesame seed meal (DSSM) obtained after supercritical CO_2_ extraction was subsequently utilized for the preparation of sesame seed protein isolate (SSPI) and sesame seed globulin (SSG). Acrylamide was obtained from ACROS ORGANICS (Geel, Belgium), bis-acrylamide was obtained from TOKU-E Company (Bellingam, WA, USA), glycine was obtained from Fisher Scientific Ltd. (Leicester, UK), sodium Lauryl sulfate was purchased from Sigma-Aldrich (St. Louis, MO, USA), Tris (molecular biology grade) was obtained from Vivantis Technologies Sdn Bhd (Selangor Darul Ehsan, Malaysia), Tricine was purchased from USB Corporation (Cleveland, OH, USA). Except stated otherwise, all chemical reagents used in this work were of analytical grade.

### 2.2. Preparation of Sesame Seed Protein Isolate (SSPI)

**Figure 1:**
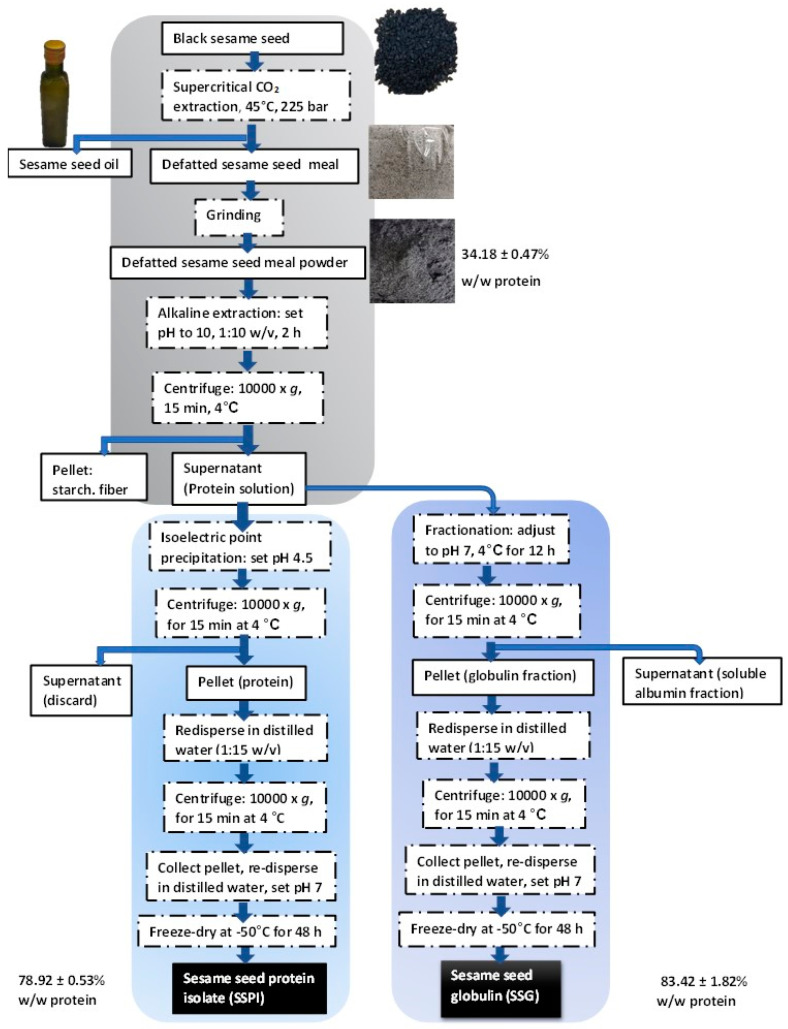
Schematic depiction of the preparation of sesame seed protein isolates via alkaline extraction and isoelectric point precipitation (AE-IP) and sesame seed globulin via alkaline extraction, fractionation, and isoelectric point precipitation (AE-F-IP).

Sesame seed protein isolate was prepared using the so-called ‘universal alkaline extraction and isoelectric point precipitation’ (AE-IP) method (Figure 1). Put succinctly, the whole defatted black sesame seed meal (DSSM) was pulverized into fine powder. DSSM powder was dispersed into distilled water at a solid-to-solvent ratio of 1:10 *w*/*v*. The pH of the dispersion was adjusted to 10.0 by adding a few drops of 2 M NaOH. The extraction was carried out by stirring for 2 h at room temperature. The mixture was then centrifuged at 10,000× *g* for 15 min at 4 °C. The supernatant was collected while the pellet was discarded. The supernatant was adjusted to pH 4.5 by adding a few drops of 5 M HCl to enable the isoelectric point precipitation of sesame seed proteins. The solution was left for 12 h at 4 °C for complete precipitation. Thereafter, sesame seed protein was collected by centrifuging the solution at 15,000× *g* for 15 min at 4 °C. The protein was collected as the pellet. The pellet was washed twice by re-dispersing it in distilled water, followed by centrifugation. Finally, the pH of the protein solution was adjusted to 7.0 using 1 M NaOH solution, frozen at −20 °C for 24 h, and freeze-dried using Labconco FreeZone 12 freeze dryer (Labconco Corp., Kansas City, MO, USA) to obtain a sesame seed protein isolate (SSPI). The coarse freeze-dried SSPI flakes were crushed into powder and stored at 4 °C.

### 2.3. Preparation of Sesame Seed Globulin (SSG)

Firstly, DSSM powder was dispersed into distilled water at a ratio of 1:10 *w*/*v*. After adjusting the pH to 10.0 using 2 M NaOH solution, the mixture was stirred at room temperature for 2 h. Then, the supernatant was collected after centrifuging at 10,000× *g* for 15 min at 4 °C. Subsequently, the pH of the supernatant was adjusted to 7.0 using a chilled HCl solution. The protein solution was kept at 4 °C for 12 h. After centrifugation of the solution, crude sesame seed globulin was collected as a pellet, whereas the supernatant, which is rich in water-soluble albumin, was discarded. The collected pellet was further re-dispersed in distilled water (containing 5% NaCl) at 1:15 *w*/*v*. The dispersion was stirred for 2 h, after which the supernatant was collected via centrifugation. The pH of the supernatant was adjusted to 4.5 by adding a few drops of 5 M HCl solution to trigger the precipitation of sesame seed globulin (SSG). After centrifugation, the SSG precipitate was collected, washed twice with distilled water, and adjusted to pH 7.0 using 1 M NaOH solution. Salt and other small molecule impurities were removed by dialyzing for 24 h against distilled water using a snakeskin dialysis membrane (MWCO: 14 kDa). The retentate was freeze-dried to obtain SSG powder, which was stored at 4 °C awaiting further use.

### 2.4. Determination of Protein Yield and Recovery Rate

The total protein content of the samples was determined using the Kjeldahl method for total nitrogen content. The protein content in the samples was then calculated using a nitrogen-to-sesame seed conversion factor of 6.25. The protein yield and protein recovery rate were determined thusly:

Protein yield (%) = [M2/M1] × 100(1)

Protein recovery rate (%) = [(M2 × P2)/(M1 × P1)] × 100(2)

where M2 and M1 are the weight in grams of sesame seed protein and defatted sesame seed meal, respectively, whereas P2 and P1 are the respective purity in percent of sesame seed protein and defatted sesame seed meal.

### 2.5. Preparation of Sesame Seed Protein Amyloid-Based Nanostructures

Amyloid fibrils from sesame seed globulin were prepared via acid hydrolysis as previously described. SSG solution (3% *w*/*w*) was prepared by dispersing the protein powder in distilled water, pH 2.0. After stirring for 2 h, the solution was kept at 4 °C for 12 h for complete hydration. The protein dispersion was then centrifuged at 10,000× *g* for 15 min at 4 °C, and the supernatant was collected. Protein aggregation was initiated by adjusting the pH of the solution to 2.0 using 5 M HCl solution followed by heating at 90 ± 5 °C while stirring at 300 rpm. Aliquots of the protein solution were collected at specific time intervals (0, 4, 8, 12, and 24 h), quenched by inserting in an ice-water bath for 20 min, and kept at 4 °C awaiting further analysis.

### 2.6. Color Analysis

The physical appearance of all the protein samples was examined using a Konica Minolta CR-400 chroma meter manufactured by Konica Minolta Optics, Inc. (Tokyo, Japan). Color parameters were recorded as L*, a*, and b* values, where the L* value represents lightness, the a* value (red/green), and the b* value (yellow/blue) [23]. The color difference, Δ*E*, was calculated as follows:

(3) $$\Delta E=\sqrt{\left[{\left({L_{0}}^{*}-{L_{1}}^{*}\right)}^{2}+{\left({a_{0}}^{*}-{a_{1}}^{*}\right)}^{2}{+\left({b_{0}}^{*}-{b_{1}}^{*}\right)}^{2}\right]}$$

where L_0_*, a_0_* and b_0_* represent the color parameters of DSSM and L_1_*, a_1_*, and b_1_* represent those of the different samples.

### 2.7. Tricine SDS-PAGE

Tricine SDS-PAGE was performed following the method outlined by [24,25] with minor modifications. Firstly, protein sample solutions were added into 1.5-mL Eppendorf tubes. GE Healthcare’s low molecular weight marker, which contained standard proteins with molecular weight in the range of 14.4–97 kDa, was prepared alongside and used as a protein marker. SDS sample loading buffer (free of β-mercaptoethanol) was added to the protein solutions, followed by mixing. Each sample of protein solution was boiled briefly (5 min) and separated on Tricine SDS-PAGE system with a 5% polyacrylamide gel stacking layer and a 12% resolving gel layer under low voltage using Atto AE-6450 Dual Mini Slab Kit for electrophoresis, Atto Corporation (Tokyo, Japan) connected to EC-105 LINE VOLTAGE 230VAC 50–60 HZ 80 W power source, E-C Apparatus Corporation (Marietta, OH, USA). Subsequently, the gels were gently removed from the glass plates, rinsed with distilled water, and immediately stained with 0.2% Coomassie brilliant blue G-250 dye in methanol: distilled water: acetic acid (45:45:10; *v*/*v*/*v*) staining solution for 12 h. After, the gels were destained with the same solvent system used in the staining solution but without the dye to enable the visualization of the protein bands of the samples.

### 2.8. Scanning Electron Microscopy Analysis

Freeze-dried protein samples were dispersed in ethanol. The samples were then spotted on an adhesive copper tape and allowed to dry. SEM micrographs of the samples were obtained using a Hitachi SU3800 scanning electron microscope (Hitachi Ltd., Tokyo, Japan) operated at 3–5 kV.

### 2.9. Transmission Electron Microscopy Analysis

TEM images of the amyloid fibrils were acquired using the JOEL JEM-2100Plus transmission electron microscope, JOEL Ltd. (Tokyo, Japan). Samples (20 µL) were initially dispersed in ultrapure water (1 mL). The protein solution (5 µL) was then carefully dropped onto the surface of the TEM grid and allowed to rest for 2 min. Afterward, the tip of a filter paper was used to gently blot off excess fluid from the grid. Then, the grid was rinsed twice, each time with a drop of ultrapure water. Subsequently, the proteins on the grid were negatively stained by dropping 10 µL of 2% uranyl acetate onto the grid’s surface. The TEM grids were allowed to air dry prior to visualization of the proteins with the microscope at 100 kV.

### 2.10. FT-IR Analysis

FTIR analysis was performed using a JASCO FT/IR-4700 spectrometer, JASCO Ltd. (W. Yorkshire, UK). Each sample powder was mixed with KBr powder and pressed into a thin translucent disc. The KBr disc was inserted into the sample holder, and spectra were captured with 120 scans at 4 cm^−1^ resolution over a range of 4000 to 400 cm^−1^. Baseline, deconvolution, and peak fitting treatments were subsequently performed using OriginPro software version 10.1.0.170, OriginLab Corporation (Northampton, MA, USA).

### 2.11. Thioflavin-T Fluorescence Spectroscopy

Thioflavin-T (ThT) stock solution (2 mM) was prepared by dissolving the powder in ultrapure water. The stock solution was filtered using a 0.22 µm syringe filter to remove any residual precipitates. ThT working solution (50 µM) was prepared from the stock solution by diluting it with ultrapure water. Then, aliquots of the protein solution (15 µL) were introduced into a 96-well microplate. ThT working solution (200 µL) was added to each protein solution and mixed well. After 10 min of reaction, the ThT fluorescence intensity of the samples was recorded using a microplate reader at an excitation wavelength of 440 nm and an emission wavelength of 480 nm. Fluorescence of ThT working solution (200 µL) with ultrapure water, pH 2.0 (15 µL) was used for background correction.

### 2.12. Surface Hydrophobicity

The surface hydrophobicity of sesame protein samples was determined via the bromophenol blue binding assay [26]. Protein dispersion (2 mg/mL) was prepared in phosphate-buffered saline (PBS), pH 6.0, by vortex mixing. Bromophenol blue dye solution (1 mg/mL) was also prepared in PBS buffer. Then, 10 mL of protein sample (2 mg/mL) was mixed with 2 mL of bromophenol blue solution. The control solution was prepared by replacing the protein solution with only PBS buffer in the protein-dye mixture. Thereafter, all samples were centrifuged for 15 min at 6000× *g,* and supernatants were collected. The supernatants were diluted 10-fold, and absorption of the samples was recorded at 595 nm.

Bromophenol blue bound (µg/mg) = 200 × [(Ac − As)/(Ac × 2)](4)

where Ac and As represent the absorbance of the control and samples, respectively.

### 2.13. Functional Attributes

#### 2.13.1. Solubility

Protein solubility was ascertained following the description given by [27] with minor modifications. Firstly, protein dispersions were prepared from the powder in a phosphate-buffered saline solution (PBS, 10 mM). The pH of the dispersions was adjusted using either 1 M NaOH or 1 M HCl to obtain dispersions with pH values from 2.0 to 10.0. Thereafter, the dispersions were stirred at 100 rpm for 2 h. After centrifuging at 8000× *g* for 20 min, the supernatants were collected. A modified Bradford assay [28] was used to determine the protein content of the supernatant (Conc. sample). The supernatant obtained from protein dispersion prepared using 0.1 M NaOH was regarded as the control (Conc. control). The protein solubility (%) was calculated thusly:

Protein solubility (%) = [Conc. sample/Conc. control] × 100(5)

#### 2.13.2. Fluid Holding Capacity

The water holding capacity (WHC) and oil holding capacity (OHC) of the protein powder samples were determined following the method described by [29]. Each protein powder (1 g) was introduced to 50-mL centrifuge tube. Distilled water (10 mL) was added to the protein sample in the case of WHC, whereas soybean oil (10 mL) was added to the powder in the case of OHC. The protein mixture was vortexed and mixed for 2 min. After 30 min, the protein dispersions were centrifuged for 20 min at 3000× *g*. The supernatants were carefully decanted to allow the weight of the wet pellet or paste to be measured. The WHC or FAC was calculated thusly:

WHC or OHC = (W2 − W1)/W0(6)

where W2 is the weight of the wet pellet or paste plus tube (g), W1 is the weight of the dry powder plus tube (g), and W0 is the weight of the dry powder (g).

### 2.14. Antioxidant Activity

The antioxidant activity of the protein samples was evaluated using DPPH and ABTS assays [30]. Protein dispersions (2 mg/mL) were prepared in distilled water. DPPH solution (0.1 mM) was prepared using methanol. The DPPH assay was performed by adding 100 µL of the protein dispersion into a 96-well microplate, followed by 100 µL of the DPPH solution. Control solution consisted of DPPH solution with distilled water. The solution mixture was mixed briefly and incubated for 30 min at room temperature in the dark. Absorbance of the samples was read at 517 nm. The antioxidant activity was presented as radical scavenging activity (RSA%).

RSA (%) = [(A_c_ − A_s_)/A_c_] × 100 (7)

where A_c_ and A_s_ in this equation represent absorbance of the control and sample, respectively.

The antioxidant activity of the protein samples was also determined using ABTS assay [8]. Firstly, the ABTS˙^+^ stock solution was prepared by mixing 5 mL of ABTS solution (7 mM) with 5 mL of potassium persulfate solution (2.45 mM). This stock solution was incubated in the dark for 12 h. The stock solution was diluted with distilled water to obtain an absorbance of 0.7 ± 0.02 as the working solution. Thereafter, protein samples (30 µL) were reacted with 250 µL of the ABTS working solution for 6 min. The absorbance of the samples was recorded at 734 nm using a microplate reader. Samples with distilled water in place of the protein solution were used as a control. Result obtained was presented as radical scavenging activity (%) calculated as described supra.

### 2.15. Biocompatibility

#### 2.15.1. In Vitro Cytotoxicity

In vitro cytotoxicity of the prepared amyloid samples was evaluated on mouse macrophage (RAW264.7) and human keratinocyte (HaCaT) cells as previously described [31] with minor modification. RAW264.7 cells were obtained from ATCC (Manassas, VA, USA), whereas HaCaT cells were obtained from CLS Cell Lines Service GmbH (Eppelheim, Germany). The cells were seeded into 96-well plates at a density of 1 × 104 cells/cm^2^ for 24 h. Thereafter, the cells were treated with SSPAN and SSGAN solution and incubated for 24 h in a CO_2_ incubator at 37 °C. Then, the exhausted media from the wells was gently aspirated. This was replaced with 200 µL fresh medium (without fetal bovine serum) containing 0.5 mg/mL MTT reagent. The plates were then incubated at 37 °C for 3 h. Subsequently, media was removed, and DMSO was added to the wells to dissolve the formazan crystals that were formed. The absorbance of the wells was measured at 560 nm using a Thermo Scientific microplate reader (Themo Scientific, Waltham, MA, USA).

Cell viability (%) = (AbsT/AbsC) × 100(8)

where AbsT and AbsC depict the absorbance of the treated sample and the absorbance of the control sample, respectively.

#### 2.15.2. Hemolytic Effect

The hemolytic effect of the protein amyloid-based nanostructures was evaluated on rat erythrocytes, according to a previous report [32]. Fresh rat blood (10 mL) was commercially obtained from the National Laboratory Animal Center, Mahidol University, Thailand. Erythrocytes were collected by centrifuging the blood at 1500 rpm for 10 min. The pellet containing erythrocytes was collected and washed thrice by resuspending in PBS, followed by centrifugation. Then, fresh erythrocyte suspension was prepared from the cell pellet (2% *v*/*v*) by adjusting with PBS. Protein dispersion was also prepared in PBS, pH 7.4. The protein dispersion (2 mL) was added to the erythrocyte suspension (2 mL) and gently mixed. The negative control consisted of PBS solution added into the erythrocyte suspension, while the positive control contained distilled water. The resultant mixture was incubated at room temperature for 30 min. The mixture was subjected to 10 min of centrifugation at a speed of 1500 rpm and at 4 °C. Supernatants of the samples were collected and diluted 4-fold with distilled water. Then, the absorption of the diluted solutions was recorded at 540 nm using a UV-vis spectrophotometer. Erythrocyte hemolysis induced by the samples was calculated thusly:

Hemolysis (%) = [(AS − AN)/(AP − AN)] × 100(9)

where AS, AN, and AP represent the absorbance of the sample, the negative control, and the positive control, respectively.

### 2.16. Statistical Analysis

All data were collected at least in triplicate. The data were analyzed using one-way ANOVA followed by the Tukey test for post hoc multiple comparisons. *p* value was set at less than 0.05 for statistical significance. All data analysis was carried out using GraphPad Prism software version 10, GraphPad Software, Inc. (San Diego, CA, USA).

## 3. Results and Discussion

### 3.1. Preparation and Characterization of Sesame Proteins from DSSM

#### 3.1.1. Protein Extraction and Recovery

In view of the growing importance of sustainability and promoting a circular economy around the world to meet the challenges posed by resource constraints, it is absolutely imperative to re-examine hitherto underexplored agri-food industry residue as a potential feedstock for the generation of value-added bioproducts and food ingredients. Herein, defatted black sesame seed meal, a highly promising protein-rich byproduct of the oil extraction process from roasted black sesame seeds using supercritical CO_2_ extraction, was examined. The purpose was to determine whether it could serve as a viable and sustainable source for the production of food proteins and amyloid nanostructures for potential use in nutritious and functional food products.

Sesame seed protein isolate (SSPI) and sesame seed globulin (SSG) fraction were successfully obtained from the defatted sesame seed meal. An overview of the workflow involved in the preparation of both SSPI and SSG is depicted in the schematic (Figure 1).

As shown in Table 1, the protein content of the defatted sesame meal powder was (34.18%). This value was within the range of crude protein content previously reported for sesame seed cake by other authors [33]. The recovery rate for both SSPI and SSG using the AE-IP method was modest, approximately 50% and 40%, respectively. Considering the fact that the preparation was based on an orthodox and simple extraction technique, the recovery rate was actually commendable. This notion is buttressed by the findings from analogous studies by Yang et al., wherein extraction of proteins by AE-IP technique from cold-pressed sesame cake with or without ultrasonic pre-treatment exhibited recovery of 22.24–25.95% and solid yield of 15.57–18.62% [33]. The protein content (%*w*/*w*) of SSPI and SSG was equally impressive for a conventional extraction process. This is particularly notable since the source material was extracted only once and without any pre-treatment step in contrast to other reports where multiple extractions were carried out on the crude material. Often, the protein content is considered as a rough indicator of purity. Apparently, SSG had better purity compared to SSPI (protein content of 83% vs. 79%), which is credited to the additional fractionation steps included during the preparation of SSG (Figure 1). Also, it could be said that both SSPI and SSG were of comparable purity to protein isolates obtained from Turkish local beans (protein content of 80–85%) by AE-IP method [34] but of superior purity to protein proteins extracted from pea flour by salt extraction, micellar precipitation or AE-IP techniques (protein content of 73–75%) [35] and protein isolate (protein content of 77.1%) obtained from sesame meal powder AE-IP approach. Although this may not strictly be an ideal comparison given the nuances sometimes involved, such as differences in the source of the raw material as well as the extraction procedure, the results in Table broadly confirmed that the protein preparation technique applied herein was efficacious.

#### 3.1.2. Physical Appearance of Sesame Protein Samples

**Figure 2:**
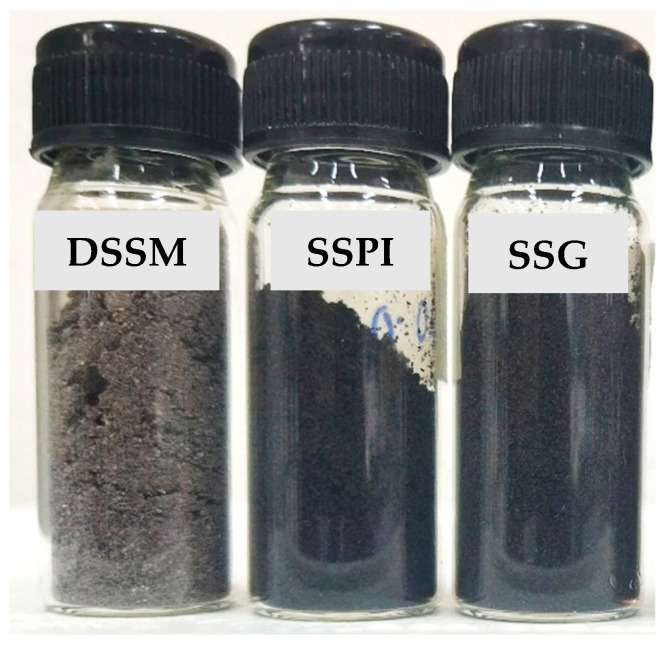
Physical appearance of defatted sesame seed meal (DSSM), sesame seed protein isolate (SSPI), and sesame seed globulin (SSG) powder samples.

The physical appearance of protein products, such as isolates, hydrolysates, or concentrates, has a major influence on consumer appeal and acceptance. This is because of the use of appearance by many as a first approximation of product quality. The visual appearance and corresponding color parameters of DSSM and protein samples prepared and derived from it are presented in Figure 2 and Table 2, respectively. It can be seen that both SSPI and SSG were similar in color but darker when compared to DSSM.

The color parameters in terms of L*, a*, and b* values were consistent with the visual appearance. The L* value denotes lightness, i.e., the higher the L* value (closer to 100), the lighter the color, and vice versa. Compared to both SSPI and SSG, the crude sesame seed flour was significantly lighter in appearance, as evinced by the higher L* value. The L* values of both SSPI and SSG did not only signify that both were darker, but also that there was no variability in their dark color. The same trend was reflected in their a* (greenness to redness) and b* (blueness to yellowness) values. With respect to the overall color, there was a large difference in the color of the protein samples, which could be visually perceived (Δ*E* value greater than 6.0) [23] relative to the DSSM sample.

Pigmentation, such as the dark color of sesame seed, had been credited to metabolites in the seed coat [18], such as flavonoids and terpenoids, as well as amino acids and their derivatives. In fact, studies have reported a greater preponderance of these valuable bioactives in black sesame seeds compared to other color varieties such as brown or white [36]. According to Dossou et al., the dark color or pigmentation is mainly due to melanin from the seed coat or hull of the sesame seed. The melanin from black sesame seed was similar to melanin from other natural sources. Polyphenols were proposed as precursors to the pigment [37]. Meanwhile, the ‘black sesame pigment’ isolated from black sesame seed was reported to exhibit strong antioxidant, reducing, and anti-nitrosating properties [38]. On the basis of the ostensible increase in the dark color of the protein samples, a proposition could be made. That is, processing the DSSM into SSPI and SSG led to the release and greater availability of bioactive pigment components ending up in the obtained protein sample. This implies that better activity of the protein samples could be obtained, thereby enriching their value as potential functional food ingredients. Meanwhile, from an aesthetic perspective, SSPI and SSG could serve an auxiliary purpose as natural colorants to improve the visual appeal of the functional food products, such as bread, cake, cookies, beverages, etc., into which they are incorporated.

#### 3.1.3. Microstructure of Sesame Seed Protein Samples

**Figure 3:**
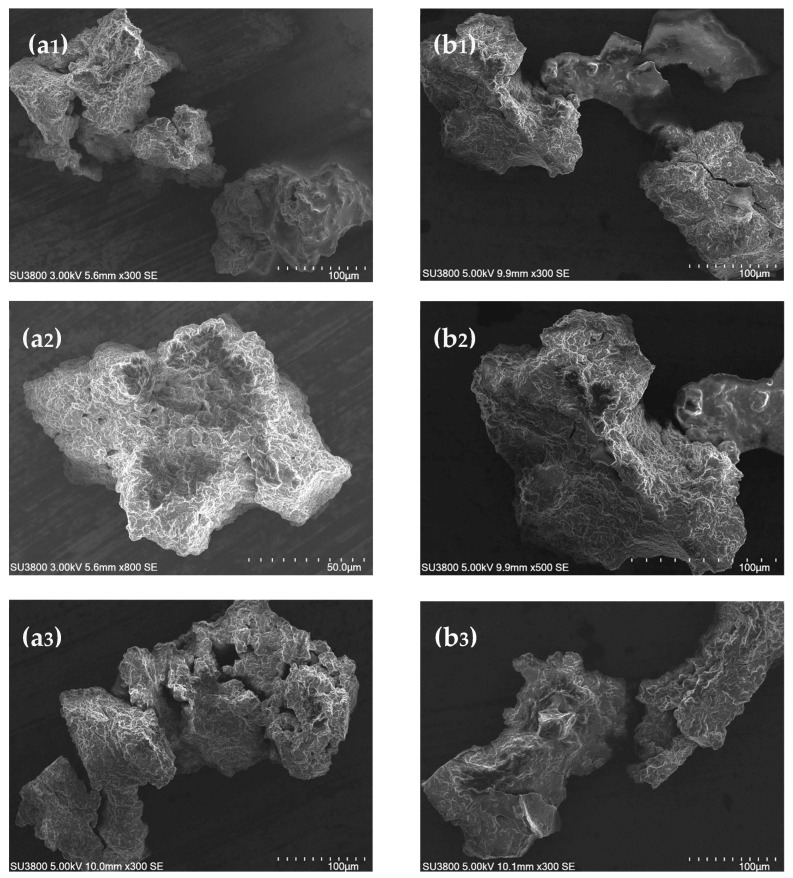
SEM images of sesame seed protein isolate, SSPI (**a1**–**a3**) and sesame seed globulin, SSG (**b1**–**b3**). Both SSPI and SSG were prepared from defatted black sesame seed meal.

The morphology and structure of sesame protein microparticles were examined using SEM. Figure 3 depicts representative micrographs of SSPI and SSG. It can be seen that particles from both samples broadly feature the same morphology, although SSG seemed a bit denser and more compact than SSPI. The protein particles manifested a rugged rock-like appearance. This quintessential morphology of sesame protein particles can be credited to the use of the freeze-drying technique during the drying of the aqueous dispersion in order to obtain solid protein samples. Freeze-drying typically takes a long time, during which ice crystals are formed. The long duration allows greater molecular interaction of the protein molecules, enabling them to coalesce and form clumps. Sublimation of the ice crystals facilitates the formation of the pores in the microstructure [39]. The ridges observed on the surface of the particles can be attributed to the rate of water loss as a consequence of sublimation during lyophilization. The rapid rate of water loss on the surface of the sample during the freeze-drying process can cause the formation of wrinkles on the surface of particles, giving rise to the ridge-like structure and rough appearance. It is apparent that the microparticles of both SSPI and SSG are irregular in shape as a result of the freeze-drying. Similar rough surfaces and non-uniformly shaped particles were observed for freeze-dried protein isolates obtained from unprocessed alfalfa seeds [40].

#### 3.1.4. Electrophoretic Mobility of Sesame Protein Samples

**Figure 4:**
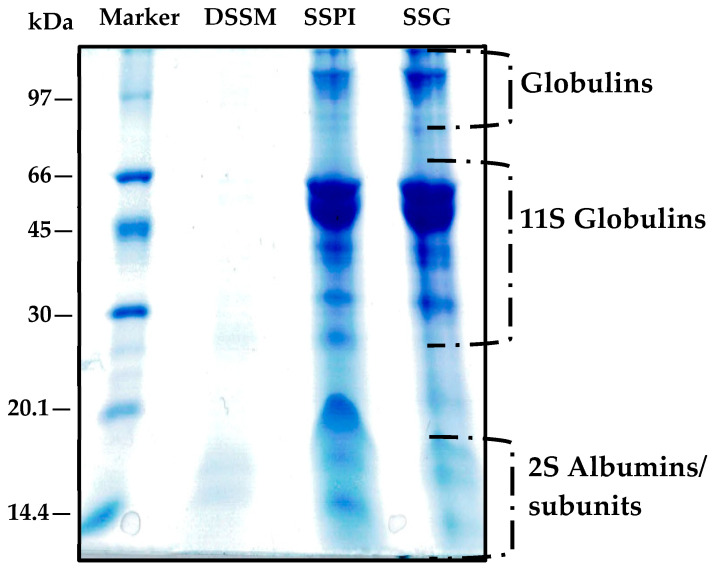
Tricine SDS-PAGE gel image of sesame proteins separated under non-reducing conditions and stained with Coomassie brilliant blue G. Marker: low molecular weight protein standard, DSSM: defatted black sesame seed meal, SSPI: sesame seed protein isolate, and SSG: sesame seed protein globulin.

The protein profile of DSSF, SSPI, and SSG were ascertained via Tricine-SDS-PAGE. Illustrated in Figure 4 is the electrophoretic pattern of the different protein samples resolved under non-reducing conditions (no β-mercaptoethanol). It can be seen that the most intense and prominent protein band was present in the range of 45–66 kDa (Figure 4). As mentioned earlier, 11S globulin makes up the majority of protein in sesame seeds, with a reported relative abundance of 78.68% [41]. The native protein exists as an oligomeric structure consisting of six subunits held together by noncovalent bonds. Each of these subunits is further composed of an acidic (30–34 kDa) and a basic (20–25 kDa) linked together by a disulfide linkage. Thus, under non-reducing (i.e., absence of disulfide bond chemical reducing agents) conditions, 11S globulin is expected to be resolved with apparent MW around 50 kDa. According to Orruño and Morgan, under non-reduced SDS-PAGE, sesame 11S globulin fraction exhibited four protein bands around 36, 41, 46.6, and 51.8 kDa [42]. The protein band around 45–60 kDa was the most intense. A similar pattern of protein bands can be observed in the SSG prepared in this, indicating that 11S globulin was the dominant component. Other authors have also noted 11S globulin in this same position under non-reducing SDS-PAGE condition. Nouska and colleagues reported an identical SDS-PAGE profile for sesame protein isolates obtained via micellization and isoelectric precipitation. The authors noted the presence of a major 11S globulin band at 50–53 kDa position when the protein isolates were resolved under non-reducing conditions. In the presence of reducing agents, the widely reported ≈20 kDa and ≈30–33 kDa polypeptides become prominent [43]. The electrophoretic pattern of 11S globulin in SSG is also similar to that of oat globulin, which presents an 11S monomer with apparent molecular weight around 55 kDa position under non-reducing conditions because of its component α (32 kDa) and β (22 kDa) polypeptides linked by a disulfide bond [44]. Meanwhile, Chen et al., using Tricine-SDS-PAGE, found that the sesame 11S globulin acidic polypeptide unit and basic polypeptide unit were located at 33 kDa and 20 kDa positions, respectively [41]. Taken together, the result seen in the present electrophoretogram suggested that the sesame 11S globulin contains disulfide bonds linking the two main polypeptide subunits, resulting in intense protein bands in the region between 45 and 60 kDa, and the prepared protein has not been altered. In addition to the 11S globulins, another protein band could be seen around position 97 kDa, which had been ascribed to sesame globulin. Other authors have also spotted this globulin fragment in sesame seed proteins at similar positions [45,46]. It has also been reported that sesame seed contains a 7S globulin, which constitutes about 1–5% of the total protein content [42,47]. Previously, the 7S globulin monomers were seen as polypeptides devoid of disulfide linkages between 12.5 and 60 kDa. The most prominent of these had a band around 45 kD [42]. Because of the low relative abundance of sesame 7S globulin, it is presumed that the protein bands around 45 kDa were overshadowed by the 11S globulin in Figure 4. Importantly, it should be pointed out that while both SSPI and SSG protein profiles were largely similar, an interesting difference emerges on closer examination. Around the 20 kDa range, it can be seen that the SSPI profile manifested an intense protein band. This protein band was less prominent in the SSG protein profile. Given that the 11S globulin basic polypeptide unit has a molecular weight of about 20 kDa, at first it might be assumed that it is representative of this band. Alternatively, it is conceivable that this protein band belongs to albumin fraction. The latter notion is supported by the absence of a disulfide reducing agent which is necessary to produce the globulin fraction at this location, thus pointing to the band being albumin. Secondly, the diminished intensity of the band in the SSG further supports that it is albumin and its lack of prominence in SSG was due to their removal during the fractionation procedure. Albumins have been previously reported to appear in similar position in the electrophoretogram of sesame protein [45] as well as in other plant proteins such as mung bean, Bambara groundnut and pea [48]. In essence, the result of the protein profiles supports the notion that the facile fractionation approach adopted herein, indeed produced a protein fraction rich in sesame globulins.

### 3.2. Formation of Sesame Protein Amyloid Nanostructures

**Figure 5:**
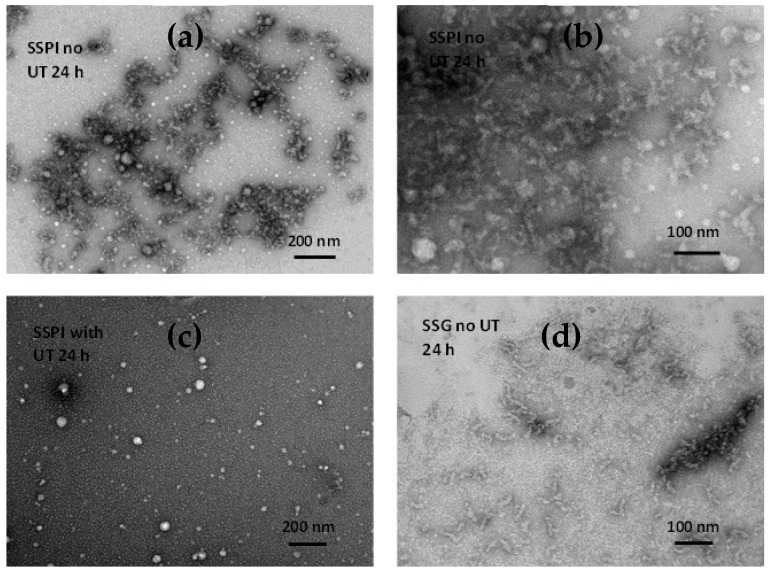
TEM images of sesame protein amyloid species after fibrillation for 24 h at pH 2 and 80 °C: (**a**) SSPI amyloid species prepared from 3% w/w protein solution without ultrasonication treatment; (**b**) Image of SSPI amyloid species at higher magnification; (**c**) SSPI amyloid species prepared from 3% *w*/*w* protein solution with ultrasonication treatment; (**d**) SSG amyloid species prepared from 3% *w*/*w* protein solution without ultrasonication treatment.

The feasibility and extent to which the prepared sesame proteins could generate amyloid nanostructures were investigated under acid-induced hydrolysis coupled with heat treatment. This condition facilitates the partial unfolding of the globular entity, release of the polypeptide chains, and cleavage into smaller peptides, which are more amyloidogenic. In this context, the tendency of the protein species to aggregate and undergo fibrillation is significantly enhanced. Insights into the morphology of the protein species following exposure to aggregation conditions after 24 h was obtained using transmission electron microscopy. The electron micrographs of SSPI and SSG after 24 h of incubation are presented in Figure 5. The TEM micrograph of SSPI revealed the presence of non-fibrillar amyloid aggregates in the form of clusters (Figure 5a,b). Some of these structures were spherical, but most of the aggregates were amorphous in shape and of varied sizes. There have been a number of reports on the protein fibrillation-enhancing effect of ultrasonication treatment [2,49]. This notion was briefly tested by applying ultrasonication treatment (power of 750 Watts, frequency of 20 kH at 50 amplitude, and pulse of 5 s ON and 5 s OFF) for 30 min at a temperature below 35 °C, CPX750 ultrasonic processor, Cole-Parmer Instruments (Vernon Hills, IL, USA) to SSPI after hydrating at pH 2.0 followed by heating. The result was somewhat interesting. Instead of the amyloid fibril aggregates that were expected, spherical amyloid nanoparticles were observed (Figure 5c). Meanwhile, the micrograph of the SSG sample subjected to aggregation conditions but without ultrasound treatment revealed aggregates with morphology that was mostly fibrillar, curly, short, and worm-like in shape (Figure 5d). The process of protein aggregation under acidic heating is an intricate phenomenon with a continuum of oligomeric intermediates having various morphologies. Kinetic and thermodynamic competition between these intermediate species would determine the predominant form of amyloid post-incubation. Additionally, many other factors could influence the amyloid formation process, including the composition of the protein isolate, the amino acid composition of the polypeptides and the hydrolyzed peptides, reaction conditions, and protein concentration, among others. Apparently, on the basis of TEM analysis, it seems SSG was more amenable to formation of amyloid with a well-defined structure relative to SSPI under the same condition. A possible explanation is that the additional fractionation steps during the preparation of SSG removed small soluble bioactive molecules, which could restrict amyloid fibril formation.

**Figure 6:**
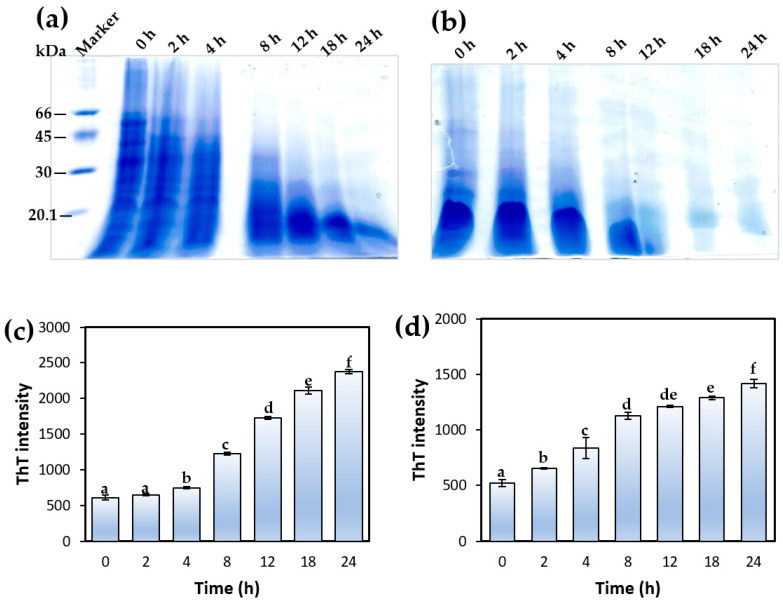
Tricine-SDS-PAGE profiles of (**a**) sesame seed globulin and (**b**) sesame seed protein isolate incubated at pH 2.0 and 90 °C depicting the gradual hydrolysis of the proteins with the increase in time (0 to 24 h). ThT fluorescence intensity of (**c**) SSG and (**d**) SSPI solutions, pH 2.0, subjected to aggregation by heating (90 °C) for 2, 4, 8, 12, 18, and 24 h. Different lowercase letters represent values that are significantly different (*p* < 0.05).

Hydrolysis of proteins into their subunits and peptides is an essential preceding step prior to heat-induced aggregation under acidic conditions (pH, 2.0) [50]. Tricine-SDS-PAGE was used to monitor the extent of SSG and SSPI hydrolysis during the course of the aggregation process (Figure 6a,b). Gel images of both SSG and SSPI aliquots during aggregation were resolved under non-reducing conditions. It is noticeable from both gel images that the 11S globulin band (≈55 kDa) intensity became narrow or greatly depleted at time 0 h in comparison to the same band for the native proteins that were not subjected to aggregation (as shown in Figure 4). This reduction in 11S globulin band intensity at time 0 h revealed that even before thermal treatment, a marked amount of the 11S globulin in SSG was hydrolyzed merely by exposing the protein solution to acidic conditions (pH, 2.0). A similar pattern was observed for SSPI, albeit the hydrolysis of 11S globulin appears to be greater. The 11S globulin from sesame seed is known to be structurally similar to the typical 11S globulin from seed proteins such as soy protein. The acid-induced hydrolysis of sesame 11S globulin observed here was in accord with reports from the research of Yu et al., who noticed that incubation of soy protein isolates at pH 2.0 caused a considerable hydrolysis of the 11S globulin subunit [51]. Application of thermal treatment (90 °C) to the protein solution under acidic milieu caused a gradual hydrolysis of the major medium molecular weight proteins (>35 kDa) with time. In fact, it can be seen that all the 11S globulin had been hydrolyzed by the 8th hour in both SSG and SSPI, and by the 12th hour, most of the proteins that remained were of low molecular weight ≤20 kDa.

The evolution of amyloid nanostructures from sesame protein solution under aggregation induced by acidic heating was monitored using a ThT fluorescence assay. ThT is a small fluorescent dye with a remarkable ability for preferential binding to the cross-β-sheet rich motifs typically found in amyloid structures. This binding interaction with amyloid proteins results in increased fluorescence intensity [52]. Thus, the ThT assay has become a valuable probe of choice for examining the presence and extent of protein amyloid formation based on its variation in fluorescence intensity [8,52,53]. The ThT fluorescence intensity of SSG and SSPI solutions at pH 2.0 subjected to aggregation under different heating times (0–24 h) is presented in Figure 6c,d. As shown in Figure 6, there was a marginal but insignificant increase in the ThT fluorescence intensity of the SSG solution after heating for 2 h at pH 2.0. This is probably an indication of the relatively modest content of β-sheet structures in the protein solution at that time because protein hydrolysis was still quite limited, and the rate of self-assembly of the peptides to amyloid species was low. In other words, the SSG aggregating solution was at a lag phase. A marked increase in the ThT fluorescence intensity of the SSG solution was observed after heating for 4 h at pH 2.0, signifying a relative increase in the content of β-sheet structures and amyloid formation. This increase in ThT fluorescence intensity was progressively enhanced with an increase in heating time from 4 to 24 h. This can be credited to the fact that the protein aggregation solution entered the so-called ‘growth phase’ where the rate of formation of amyloid species with characteristic β-sheet structures from self-assembly of the peptides increased considerably [54]. In the case of the SSPI solution at pH 2.0, the lag phase appeared to be shorter, as evinced in the substantial increase in fluorescence intensity after heating for 2 h. The increase in ThT fluorescence intensity continued from 2 to about 8 h, suggesting an increasing rate of formation of β-sheet structure as a consequence of protein aggregation. Beyond 12 h of heating, the increase in ThT fluorescence intensity was less dramatic, which may be due to the protein aggregation reaction entering the stationary phase [53]. At this point, the content of peptides in the solution available for self-assembly is lower, which diminishes the vigor of the aggregation reaction. In both protein solutions, the ThT fluorescence intensity following heating from 4 to 24 h was clearly higher than the solution without heating, underscoring the progressive formation of amyloid nanostructures with thermal treatment of the protein solutions at acidic conditions (pH 2.0). A similar increase in the ThT fluorescence intensity of rice glutelin solution (pH 2.0) was noted by Li et al. after heating for 24 h, confirming the formation of amyloid-based nanostructures [8]. It is worthy of note that the impact of heating on formation of amyloid nanostructures was varied between both SSG and SSPI samples, perhaps due to difference in the unfolding transition temperature of both samples resulting in slightly different ThT fluorescence intensity pattern (Figure 6). Indeed, it has been previously noted that the unfolding transition of proteins subjected to aggregation is contingent to the mode of sample preparation, ionic strength of the medium, structure of proteins as well as modification of the proteins [11]. The result from the ThT fluorescence assay of sesame protein aggregation indicated a progressive increase in protein amyloid formation with increase in heating time under acidic condition. When this result is considered in light of the different morphologies revealed by TEM, the ThT result could be an indication that there may be an optimal heating time for obtaining amyloid nanostructures with different features.

### 3.3. Secondary Structure Analysis of Sesame Proteins and Amyloid Nanostructures

**Figure 7:**
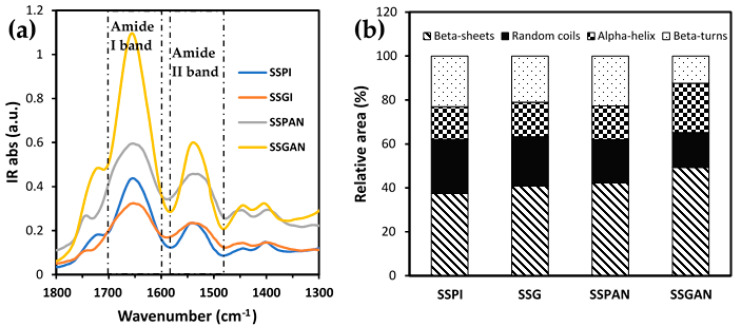
(**a**) FTIR spectra of sesame proteins (SSPI and SSG) and amyloid-based nanostructures (SSPAN and SSGAN) revealing noticeable peaks in the Amide I and Amide II regions; (**b**) relative content of secondary structure in the Amide I band obtained from the relative area of corresponding Gaussian components, i.e., size of each resolved peak in percent.

Further insights pertaining to the secondary structure and likely conformational alterations of the protein isolates and amyloid nanostructures were facilitated with the aid of FTIR analysis. Figure 7 depicts the FTIR spectra of the protein samples in the region 1800–1300 cm^−1^, which encompasses the Amide I band located between 1600 and 1700 cm^−1^ and the Amide II band delineated by IR absorption from 1580 to 1480 cm^−1^. The full FTIR spectra (4000–400 cm^−1^) are included in Supplementary Materials (Figure S1). The Amide I band represents the stretching vibration of C=O bond (80%), whereas the Amide II band corresponds to the vibrations emerging from a combination of the C–N bond stretching (30%), N–H bond bending (60%), and C–C bond stretching [55]. The strong IR absorption peaks noticeable in the Amide I and II spectral regions (Figure 7) are a testament to the proteinaceous nature of the sesame samples. The peak position of SSPI (1653 cm^−1^) and SSG (1652 cm^−1^) Amide I bands was similar (1654/1655 cm^−1^) to those obtained by other authors for sesame protein isolate [33]. By dint of its exceptional signal-to-noise ratio and remarkable sensitivity to protein secondary structure, the Amide I (1700–1590 cm^−1^) band of each sample was used for evaluating the composition of the various secondary structural elements present in the proteins pre and post-aggregation. This was accomplished by deconvolution of the Amide I band into component peaks. Peak position and assignment were in accordance with insights drawn from previous studies. The corresponding peak area as a percent of the total area of all peaks in the Amide I region was used to calculate the composition the four major structural elements, namely β-sheets (1618 cm^−1^, 1625 cm^−1^, and 1634 cm^−1^, 1680 cm^−1^), random coils (1644 cm^−1^), α-helix (1651 cm^−1^ and 1658 cm^−1^), and β-turns (1667 cm^−1^) [43,55,56].

In all the samples, β-sheets emerged as the most abundant secondary structural element. The difference in the relative composition of the secondary structure of the native proteins, when contrasted with those subjected to aggregation, attests to the fact that the proteins were structurally modified by the aggregation process. Among the various samples, it was noticed that the content of β-sheets increased remarkably after the proteins were subjected to aggregation treatment. Meanwhile, the composition of random coils decreased following the acidic heating of the native proteins. This observation is consistent with the notion that the protein conformation became well-organized and ordered after aggregation. This was more pronounced in the case of SSGAN, which exhibited greater content of β-sheet structures compared to SSPAN. The increased content of β-sheet in SSGAN and conformational re-alignment support the presence of amyloid structures in the sample.

### 3.4. Solubility and Surface Hydrophobicity

Solubility is a critical attribute of vital importance in the application of proteins in food. The protein solubility could profoundly impact essential aspects such as the foaming, gelation, emulsification, color, texture, and sensory attributes of the protein-incorporated food product. One factor that greatly impacts protein solubility is its hydrophilicity/hydrophobicity balance, which in turn is dependent on the composition of the surface amino acids. In a case where there is a greater number of surface hydrophilic amino acid residues and a higher net charge leading to greater electrostatic repulsion and ionic hydration, the tendency of the protein is one of higher solubility and vice versa [14].

**Figure 8:**
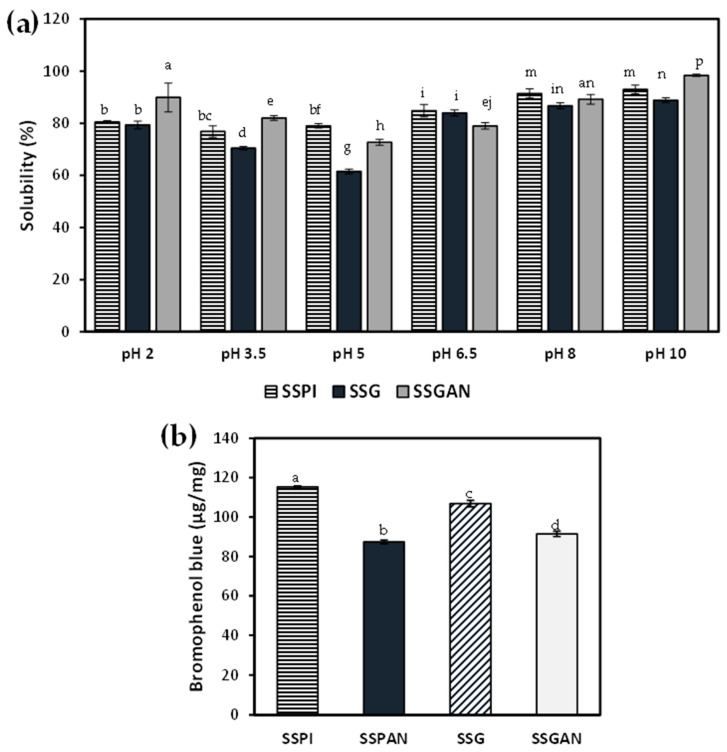
(**a**) Solubility of sesame protein isolate, globulin, and amyloid fibrils under different pH conditions; (**b**) Surface hydrophobicity of sesame protein samples. Different lowercase letters (e.g., a, b, c) on the columns represent values that are significantly different from each other (*p* < 0.05).

Apparently, protein solubility is pH-dependent. Solubility of both SSPI, SSG, and SSGAN from pH 2.0 to 10.0 is presented in Figure 8a. Limited availability of SSPAN samples meant that solubility could only be performed on SSGAN. The solubility of all protein samples was at least around pH 3.5–5.0, which is consistent with the pH range of the isoelectric point of sesame proteins. In general, there was an increase in solubility toward the alkaline pH range, which is from 6.5 to 10. This is consistent with previous reports. Also, there appears to be an increase in solubility of the proteins, especially SSGAN, at acidic pH (2.0) and alkaline pH (8.0–10.0), which is valuable for food product formulation. SSGAN was significantly more soluble than SSPI and SSG at pH 2.0–3.5 (*p* < 0.05). The increase in SSGAN solubility at low pH can be explained in part by the fact that at low acidic pH, amyloid samples typically present greater positively charged structures compared to their native monomers [4,8,57]. This increased net positive charge engenders greater electrostatic repulsion of the amyloid species, thereby obviating their precipitation in solution. Consequently, the physical stability of the amyloid suspension is enhanced [8]. Also, the presence of hydrolyzed peptides with small molecular weights in the amyloid sample can improve the solubility of SSGAN at low pH. It has been reported that the extraction method or processing could have a considerable impact on the solubility of obtained products such as protein isolate, concentrate, etc. For instance, it was observed that the processing of raw sesame meals by cooking, microwave, or ultrasound improved the protein solubility of the processed sesame meal relative to its raw counterpart [14]. It can, therefore, be inferred from the result herein that modification of SSG via amyloid formation can improve the protein solubility in an acidic milieu.

Understanding the surface hydrophobicity of plant-based proteins and protein amyloid species is important because hydrophobic interactions have a considerable impact on protein conformation, structure, as well as functional characteristics such as the proteins’ affinity toward the water–oil interface. To obtain insights with respect to the surface hydrophobicity of the proteins, bromophenol blue dye was used as a probe. That is, the higher the amount of bromophenol blue bound to the protein, the higher the surface hydrophobicity. Data on the surface hydrophobicity are shown in Figure 8b. The results revealed that in both SSPI and SSG, the formation of amyloid-based nanostructures after heating for 24 h at pH 2.0 markedly reduced the surface hydrophobicity. It has been reported that at the initial heating period of the aggregation process (≈2–4 h), surface hydrophobicity typically increases due to protein hydrolysis coupled with partial unfolding of the protein structure [51]. As a result, hydrophobic amino acid residues, which are normally buried inside the globular protein, become exposed to the surface. The exposed hydrophobic patches on the protein surface interact, causing self-assembly and the formation of aggregate species or protofibrils. Interestingly, extending the heating time has proven to not only promote protein aggregation into amyloid nanostructures but also decrease the surface hydrophobicity of the obtained amyloid species. The latter is attributed to the reburial of the hydrophobic regions rich in β-sheets as they re-assembled to form ordered amyloid structures [58]. So, while it is common to see an increase in surface hydrophobicity of some plant proteins post-formation of amyloid species, it is not uncommon to also see the reverse in others such as chicken pea, lentil, and pumpkin seed protein isolates [4]. The result obtained from this study concurred with a similar work by Xu et al., who also noticed that the surface hydrophobicity of oat globulin decreased substantially after heating for 24 h at acidic conditions because of the formation of oat globulin fibrils [10].

### 3.5. Water Holding and Oil Holding Capacity of Sesame Protein and Amyloid Structures

**Figure 9:**
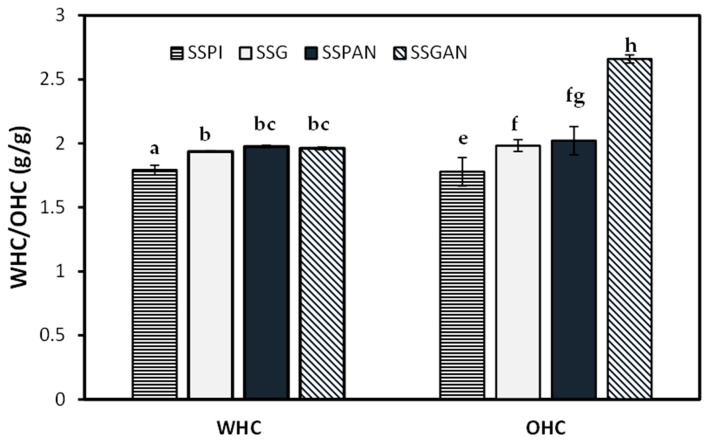
The water holding capacity (WHC) and oil holding capacity (OHC) of sesame seed protein isolate (SSPI), sesame seed globulin (SSG), as well as their respective amyloid nanostructures, SSPAN and SSGAN. Different lowercase letters (e.g., a, b, c) on the columns represent values that are significantly different from each other (*p* < 0.05).

The water absorption capacity of the protein samples is represented in Figure 9. It can be observed that the water retention ability of the sesame protein amyloid nanostructures was higher than that of the native proteins. It is understood that for proteins subjected to thermal-mediated unfolding under acidic conditions, there is an increase in the reactive sulfhydryl groups, the distribution and number of charges, as well as the hydrophilic and hydrophobic groups. This increases the tendency and potential of the protein functional groups to form strong intermolecular interactions with water molecules [59]. Besides the obvious fibrillar species in the amyloid nanostructures, non-fibrillar and peptides resulting from acid-mediated hydrolysis of the polypeptides are also present as part of the amyloid sample [56]. These peptide species tend to be more hydrophilic and, therefore, contribute toward increasing the water holding capacity of the amyloid nanostructure [59]. Meanwhile, it is understood that protein fibrillation increases the overall charge of the fibrillated protein relative to the native proteins [57], and this increase in charge improves the ability of the amyloid protein sample to interact with water molecules.

With respect to the oil absorption capacity of the protein samples, the trend was positive following amyloid formation, i.e., the OHC increased markedly for the aggregated proteins relative to their native counterpart. Protein aggregation is enabled by the unfolding of the compact globular proteins due to the thermal treatment that exposes the hydrophobic residues and polypeptide chains, which become converted into various oligomeric and fibrillar species. As such, the surface area for hydrophobic interaction between protein species and monolayer oil is increased in the amyloid protein compared to native protein. This leads to a greater amount of oil binding and retention by the amyloid nanostructure. As such, the sesame amyloid nanostructures could be valuable in the preparation of food products with high fluid holding requirements.

### 3.6. Antioxidant Activity of Sesame Protein Amyloid Nanostructures

Oxidation of food can lead to a significant decline in quality, resulting in poor taste, aroma, flavor, texture, and color, as well as enrichment with undesirable and unhealthy metabolites such as free radial and reactive oxygen species. Thus, fortifying and increasing the antioxidant content of food products have become common place as a valuable strategy to mitigate oxidative deterioration, preserve food quality, and enhance shelf-life. Increasingly, consumer preference has been shifting toward the use of natural antioxidants for food application. Plant proteins in their various forms and compositions, including concentrates, hydrolysates, and peptides, have been reported for their antioxidant activity. Interestingly, amyloid fibrils from food proteins are increasingly being recognized for having appreciable radical scavenging properties.

**Figure 10:**
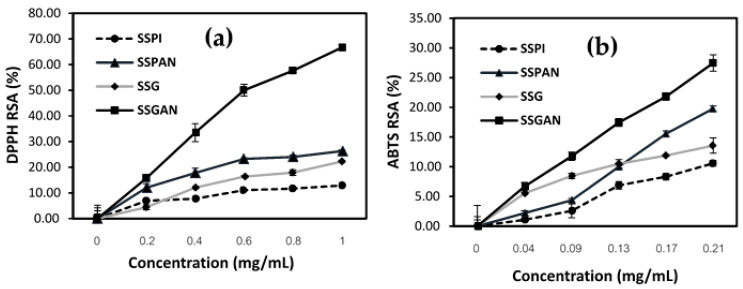
Radical scavenging activity of sesame seed proteins and amyloid nanostructure against the following: (**a**) DPPH; (**b**) ABTS radicals.

As evidenced in the results from DPPH and ABTS assays (Figure 10), the antioxidant activity of sesame proteins was remarkably increased after the formation of amyloid nanostructures. This finding is consistent with results from other studies. For instance, Mohammadian et al. found that the antioxidant property of amyloid fibrils derived from whey protein isolate and hydrolysate was substantially higher than that of their respective precursor proteins. Meanwhile, Li et al. established that fibrillization of rice glutelin markedly elevated the antioxidant activity of the obtained amyloid nanostructures [60]. As often noted, early events of protein amyloid formation under elevated temperature and acid pH are characterized by partial unfolding and hydrolysis of the native proteins into amyloid-competent peptides. Du et al. [20], in their study, also found that the antioxidant activity increased following enzymatic hydrolysis and unfolding of black sesame seed protein. The antioxidant properties of protein amyloid species have been credited to their peptide building blocks. Wei et al. noted that the antioxidant activity of ovotransferrin amyloid fibril prepared via heating at 90 °C in acidic milieu (pH 2.0) was due to its constituting peptides [61]. Indeed, it has been previously noted that antioxidant property is one of the common features shared by both peptides in amyloid structures and bioactive peptides [62]. Compared to their precursor proteins, the peptides in these structures tend to offer greater solvent accessibility of their active amino acid residues—the main driver for the antioxidant activity [63].

Amino acid resides such as methionine, cysteine, phenylalanine, tryptophan, tyrosine, and histidine are among the most active in terms of antioxidant properties. A good number of these residues, including tyrosine and tryptophan, are not only typically found in amyloid structures where they participate in π–π stacking [64], but also in sesame seed proteins [14]. The antioxidant activity of these amyloid structures could be modulated via a number of mechanisms, such as chelating metal ions, scavenging free radicals and reactive chemical species, or participating in the breakage of free-radical chain reactions. The antioxidant activity of sesame proteins and amyloid nanostructures is a testament that the amyloid nanostructures could be used as ingredients in enhancing food quality by inhibiting oxidation as well as in the development of functional foods.

### 3.7. Biocompatibility of Sesame Protein Nanofibrils

A fundamental and cogent consideration in the production and utilization of protein amyloids is their safety as food ingredients for nutrition. Unlike native food proteins, which have been part of human diets from time immemorial without many worries besides the occasional incidence of allergenicity, protein amyloids derived from food proteins have been restricted in their application as food ingredients for nutrition and health. The reluctance in use of food protein amyloids is linked to the historical association of amyloids in general to the development of many chronic and neurodegenerative diseases. Notable examples of this include the polypeptide hormone amylin, which is released together with insulin as a response to food. Aggregation of amylin causes the protein to suppress the activity of insulin and glucagon, leading to type 2 diabetes mellitus [65]. Also, β-amyloid peptide and α-synuclein aggregation and amyloid formation have been linked to the development of Alzheimer’s disease and Parkinson’s disease, respectively. Specifically, with respect to the safety of amyloids derived from food proteins, a consensus is yet to emerge since some studies indicate that they (e.g., egg-white lysozyme) are toxic [66], whereas others deem them (soy, whey, egg-white, kidney, and bean amyloid) as safe [67]. In this context, to obtain clarity as to whether sesame-derived amyloids are safe or potentially harmful, in vitro biocompatibility studies were performed.

**Figure 11:**
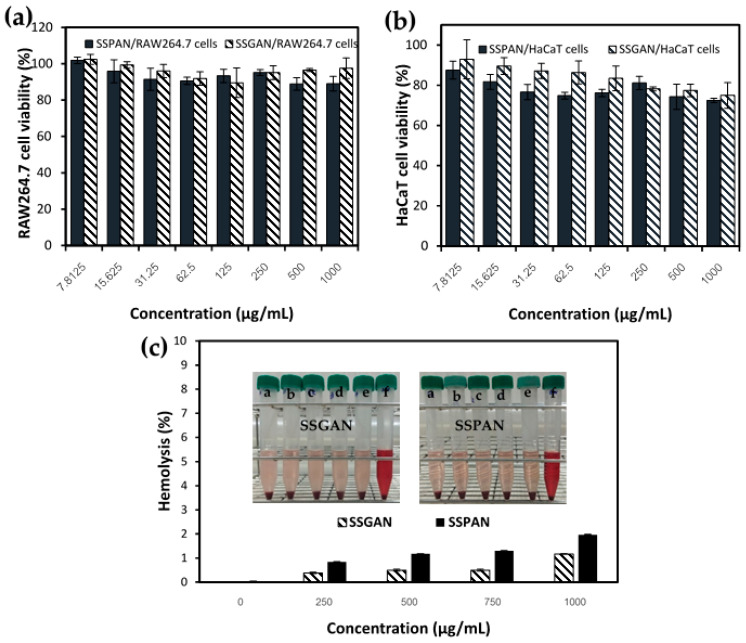
Effect of SSGAN and SSPAN on viability of (**a**) murine macrophage RAW264.7 and (**b**) human keratinocyte HaCaT cells 24 h after treatment; (**c**) Hemolytic effect of SSGAN and SSPAN on rat red blood cells (insert depicts image of the sample treated erythrocyte suspension after centrifugation at 1500 rpm for 10 min. a–e represent samples treated with 0–1000 µg/mL protein amyloid, whereas f connotes sample treated with distilled water only).

Figure 11 represents the results of in vitro cytotoxicity and hemolytic effect of SSPAN and SSGAN. As shown in Figure 11a, following the exposure of murine macrophage (RAW264.7) cells to the sesame amyloids (7.81–1000 µg/mL) for 24 h, there was a noticeable decrease in the cell viability to about 90% following exposure of cells to both amyloid samples at a concentration of 250 µg/mL. At higher concentrations of SSPAN and SSGAN (up to 1000 µg/mL), the cell viability was further reduced by not lower than 88%. A similar decrease could also be seen in the cell viability of human keratinocyte (HaCaT) cells following treatment with the amyloid samples. In both cell lines, it can also be seen that the cells were marginally more viable in the presence of SSGAN compared to SSPAN. Importantly, the viability at all treatment concentrations remained high (greater than 75% in HaCaT cells and 80% in RAW264.7 cells), suggesting that the amyloids were non-toxic to the cells [68]. Furthermore, the results on erythrocyte hemolysis show that for erythrocytes exposed to either SSPAN or SSGAN (250–1000 µg/mL), the percentage hemolysis was less than 2% (Figure 11b), which is below the threshold for materials which do not possess any hemolytic activity (that is hemolysis value <5%) [69]. From these findings, it can be inferred that the amyloids prepared from SSG and SSPI were non-toxic to the treated cells. A similar observation was also made by Lassé and co-researchers, who found that amyloid fibrils obtained from food proteins, namely kidney bean, whey, egg white, and soy protein isolates, were non-toxic to Hec-1a and Caco-2 cell lines in vitro [67]. Further credence on the safety of food amyloid was offered by a recent study in which the authors demonstrated that in vitro digested amyloid fibrils from milk β-lactoglobulin and hen egg-white globulin were non-toxic in both in vitro and in vivo models. In fact, the digested amyloid fibrils promoted cell viability in Caco-2 and HCEC cell lines because they served as nutrients to the cells [1]. Based on the result obtained in the present work, it is apparent that SSPAN and SSGAN are biocompatible and, thus, are potentially safe for use as ingredients for possible advancement of nutrition and health.

## 4. Conclusions

The self-assembly of plant proteins into amyloid-based structures presents an interesting modification strategy for improving their functionalities and extending their potential application in food and healthcare. Up until now, proteins derived from sesame seeds have yet to be investigated as potential amyloid materials. So, in this work, the viability of defatted sesame seed meal was explored as an inexpensive, abundant, and sustainable source of plant-based proteins and amyloid structures. We demonstrated that sesame seed protein fraction rich in 11S globulin could be facilely prepared from DSSM. Under acid-induced hydrolyses and heating, the sesame seed globulin was converted into fibrillar amyloid nanostructures with high β-sheet content via self-assembly of the hydrolyzed peptides. Importantly, the prepared amyloid nanostructure from sesame seed globulin exhibited improved solubility at low pH, oil retention, and radical scavenging properties. In addition, the sesame protein amyloid preparation demonstrated a good safety profile. In aggregate, these findings indicate that amyloid-based nanostructures derived from sesame seed globulin could be a promising ingredient for application in functional foods and plant-based food products. It also highlights the fact that proteins and amyloid preparation could be a valuable strategy for upcycling and adding value to defatted sesame seed meal. In the future, the sesame seed globulin amyloid nanostructures could be further exploited in the delivery of valuable nutraceutical ingredients, in tailoring the functional properties of foods, such as gelation, fluid holding capacity, interfacial properties, as well as in the development of edible food coatings and scaffolds for plant-based meat analogs.

## Figures and Tables

**Table 1:** Protein extraction, yield, and recovery rate of defatted black sesame seed protein samples.

Sample	Protein Content (%)	Yield (%)	Recovery Rate (%)
DSSM	34.18 ± 0.47 ^a^	-	-
SSPI	78.92 ± 0.53 ^b^	21.53 ± 1.22 ^c^	49.71 ± 0.98 ^b^
SSG	83.42 ± 1.81 ^c^	16.33 ± 0.91 ^d^	39.86 ± 0.76 ^a^

Different superscript letters within the same column represent mean values that are significantly different (*p* < 0.05).

**Table 2:** Colorimetric parameters of defatted black sesame seed protein samples.

Sample	L*	a*	b*	Δ*E*
DSSM	44.92 ± 0.31 ^a^	0.40 ± 0.05 ^c,d^	5.02 ± 0.08 ^a^	-
SSPI	33.39 ± 0.07 ^b^	0.11 ± 0.04 ^c,d,e^	4.40 ± 0.40 ^b^	11.66 ± 0.37 ^a^
SSG	33.24 ± 0.29 ^b^	−0.38 ± 0.01 ^c,e^	3.37 ± 0.05 ^c^	11.83 ± 0.32 ^a^

Different superscript letters within the same column represent mean values that are significantly different (*p* < 0.05).

## Data Availability

The original contributions presented in the study are included in the article, further inquiries can be directed to the corresponding author.

## Supplementary Materials

The following supporting information can be downloaded at https://www.mdpi.com/article/10.3390/foods13142281/s1, Figure S1: FTIR spectra of sesame proteins (SSPI and SSG) and amyloid-based nanostructure (SSPAN and SSGAN) from 4000–400 cm^−1^ highlighting prominent peaks.
