# Supplementary material for: Upcycling of Defatted Sesame Seed Meal via Protein Amyloid-Based Nanostructures: Preparation, Characterization, and Functional and Antioxidant Attributes
Source: Foods. 2024 Jul 20;13(14):2281. doi: 10.3390/foods13142281 (PMC11276470; doi:10.3390/foods13142281)
Supplement: Supplementary file 1 [file foods-13-02281-s001.zip › foods-3098506-supplementary.pdf]

Supplementary Material

Upcycling of defatted sesame seed meal via protein amyloid-based nanostructures: Preparation, characterization, functional and antioxidant attributes

Fredrick Nwude Eze[1,2], Ratanna Muangrat[2,3]*, Sudarshan Singh[4], Wachira Jirarattanarangsri,[2] Thanyaporn Siriwoharn[2] and Yongyut Chalermchat[2]

1 Office of Research Administration, Chiang Mai University, Chiang Mai 50200, Thailand.
2 Faculty of Agro-Industry, Chiang Mai University, Chiang Mai 50100 Thailand.
3 Department of Food Process Engineering, Faculty of Agro-Industry, Chiang Mai University, Chiang Mai 50100, Thailand.
4 School of Medical & Allied Sciences, K.R. Mangalam University, Gurugram 122103, India. Email: sudarshansingh83@hotmail.com
* Correspondence: rattanamuangrat@yahoo.com, rattana.m@cmu.ac.th

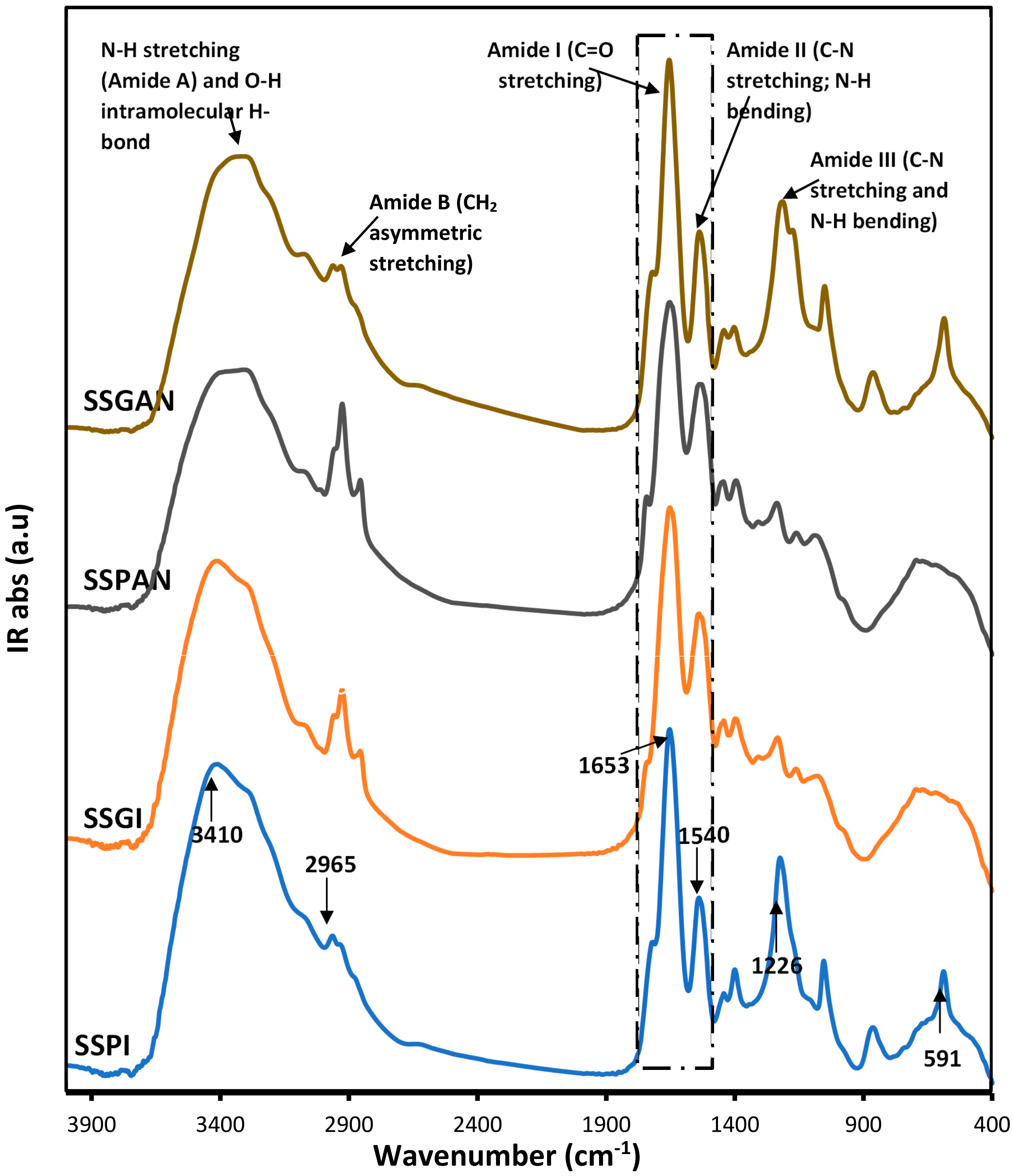

Figure S1. FTIR spectra of sesame proteins (SSPI and SSG) and amyloid-based nanostructure (SSPAN and SSGAN) from 4000 – 400 cm^{-1} highlighting prominent peaks.
